# Neural Stem/Progenitor Cells Regulate Neuroinflammation: Mechanisms and Therapeutic Applications in Neurological Diseases

**DOI:** 10.3390/ijms27094078

**Published:** 2026-05-02

**Authors:** Xu Liu, Aikun Liu, Yue Li, Yuchao Guo

**Affiliations:** 1 College of Pharmacy, Qilu Medical University, Zibo 255300, China; liuxu@qlmu.edu.cn (X.L.);; 2 State Key Laboratory of Mechanism and Quality of Chinese Medicine, Institute of Chinese Medical Sciences, University of Macau, Macau 999078, China; 3 Department of Pharmaceutical Sciences, Faculty of Health Sciences, University of Macau, Macau 999078, China; 4 State Key Laboratory of Component-Based Chinese Medicine, Institute of Traditional Chinese Medicine, Tianjin University of Traditional Chinese Medicine, Tianjin 301617, China

**Keywords:** neuroinflammation, neural stem/progenitor cell, neurological diseases, microglia, interleukin, tumor necrosis factor-α

## Abstract

Neuroinflammation plays a critical role in the pathogenesis of various neurological diseases. Therefore, alleviating neuroinflammation has become a core therapeutic strategy for these disorders. In recent years, neural stem/progenitor cell (NSPC) transplantation has shown remarkable advantages and broad prospects in the treatment of neurological diseases. This narrative review systematically summarizes research progress over the past decade on how NSPC transplantation ameliorates neurological deficits by regulating neuroinflammation-related signaling pathways, and compares the shared mechanisms and disease-specific differences. In addition, we discuss the key bottlenecks limiting the clinical translation of NSPC transplantation and propose future research directions. Accumulating preclinical evidence highlights NSPC transplantation as a compelling candidate intervention for multiple neurological disorders.

## 1. Introduction

Nervous system inflammation, also termed neuroinflammation, is a cellular immune response characterized by the activation of glial cells and the production of proinflammatory factors [1]. As a natural defense mechanism against acute events such as infection and injury, it first aids in damage control, debris clearance, and the initiation of healing [2]. However, persistent or excessive inflammation may result in pathological damage and is closely associated with the onset and progression of multiple neurological diseases, including Alzheimer’s disease (AD), Parkinson’s disease (PD), and multiple sclerosis (MS) [3]. Therefore, modulating the targets and mechanisms of neuroinflammation may be an effective approach for the prevention and treatment of neurological diseases.

Neural stem/progenitor cell (NSPC) transplantation has emerged as a promising therapeutic strategy for neurological diseases. Exogenous NSPC transplantation reshapes the injured central nervous system (CNS) microenvironment via neuroinflammation regulation to ameliorate cognitive deficits, with core therapeutic advantages in paracrine effects and intercellular crosstalk rather than mere cellular replacement [4,5]. In addition, transplanted NSPCs exert multiple reparative effects within the CNS, including promoting neurogenesis and restoring functional connectivity, thereby mediating therapeutic benefits against neurological disorders [6]. NSPCs are characterized by their self-renewal ability and their potential to differentiate into multiple types of neural cells. Beyond replacing dead brain cells, they contribute to the formation of a microenvironment that supports host cell survival and normal CNS function, while also preserving blood–brain barrier (BBB) integrity [7].

This is a narrative review that aims to systematically summarize the mechanistic advances and therapeutic potential of NSPCs in regulating neuroinflammation, with a particular emphasis on their effects on neurological disorders. A targeted literature search was performed in PubMed, with the scope primarily restricted to studies published from 2016 to 2025 to focus on the latest mechanistic and preclinical research advances in this field. The literature selection was based on the following criteria: (1) original research articles with in vivo/in vitro experimental data on NSPCs and neuroinflammation; (2) seminal review articles that summarize the core progress of the field; (3) studies published in English with full-text availability. The exclusion criteria were non-experimental studies and studies without direct relevance to NSPC-mediated neuroinflammation regulation. This screening strategy ensures the focus and novelty of this narrative review, which centers on the latest fundamental mechanisms underlying neuroinflammation regulation by NSPC transplantation. It aims to identify core cross-disease molecular pathways and establish a theoretical basis for clinical translation, rather than providing a systematic assessment of clinical efficacy. Notably, it is the first to integrate the common mechanisms and disease-specific differences in neuroinflammation regulation by NSPCs of different sources in neurological diseases, outline supporting strategies and translational bottlenecks of NSPC transplantation, and provide an integrated framework for mechanistic optimization and clinical translation of NSPC-based therapy.

## 2. Neuroinflammation

In response to the microenvironment, astrocytes and microglia exhibit changes in both morphology and function in the CNS. Following CNS injury, the interaction between these two cell types mediates neuroinflammation [8]. Microglia release a variety of cytokines and inflammatory mediators as signaling molecules, facilitating communication between microglia and astrocytes [9].

Microglia function as the resident immune cells within the CNS, protecting it from potential pathogens and pathological damage. They secrete either pro-inflammatory or anti-inflammatory cytokines based on the environmental context. However, microglial activation does not follow a simple M1/M2 dichotomy; instead, it exhibits a highly heterogeneous and dynamic phenotypic spectrum. Regulated by microenvironmental signals, microglia can adopt a variety of states, ranging from pro-inflammatory to anti-inflammatory [10]. Pro-inflammatory microglia can release tumor necrosis factor-α (TNF-α), interleukin (IL)-6, and IL-1β, thereby exacerbating neuroinflammation [11]. In contrast, anti-inflammatory microglia secrete factors such as IL-10, transforming growth factor-β (TGF-β), and insulin-like growth factor-1 (IGF-1) to suppress inflammation and promote neurogenesis. Moreover, the two phenotypes are interconvertible rather than undergoing irreversible polarization [12,13]. For the convenience of subsequent description, we hereafter refer to the pro-inflammatory phenotype as the M1 type and the anti-inflammatory phenotype as the M2 type throughout this review. Therefore, facilitating the shift in microglial polarization from M1 pro-inflammatory to M2 anti-inflammatory phenotypes could be a promising therapy for neurological disorders. The phenotypic heterogeneity of microglia is closely associated with metabolic reprogramming and epigenetic modifications [14], and their paracrine crosstalk with astrocytes and NSPCs constitutes a core mechanism for regulating neuroinflammation [15,16].

In an inflammatory environment caused by microglia, astrocytes release proinflammatory cytokines that disrupt the BBB, worsening neuroinflammation [17]. Astrocytes are the most numerous cell type within the CNS and interact with all brain cell populations, thereby modulating BBB function, offering metabolic support to neurons, and contributing to synaptic integrity as well as immune responses [18]. Upon CNS injury, astrocytes become activated into reactive astrocytes. Notably, when oxidative phosphorylation in astrocytes is impaired, the lipid composition of the brain is significantly disrupted, triggering a range of pathological mechanisms that lead to neuroinflammation and cognitive impairment [19]. In addition, reactive astrocytes can promote immune cell infiltration, upregulate glial fibrillary acidic protein, undergo morphological alterations and proliferation, and thereby exert complex effects on inflammation in the CNS [20,21].

Intervening in astrocytes, microglia, and their crosstalk, as well as targeting key molecules involved in cellular interactions to effectively suppress the neuroinflammatory progression, may provide better therapeutic options for numerous neurological diseases. NSPC transplantation can modulate the phenotypic transition of microglia and the activation of astrocytes via paracrine factors, thereby disrupting the pro-inflammatory positive feedback loop between these two cell types. This constitutes the core mechanism underlying NSPC-mediated neuroinflammation and represents the central focus of this narrative review.

## 3. NSPCs and Neurological Diseases

Neural stem cells (NSCs) possess the capacity for self-renewal and pluripotency to differentiate into neurons, astrocytes, and oligodendrocytes, and are mainly derived from embryos and the adult subventricular zone [22]. Neural progenitor cells (NPCs) are the progeny of differentiated NSCs, with high proliferative potential but relatively restricted differentiation fates [22]. NSPCs are a collective term for NSCs and NPCs and serve as the focus of this review. NSCs/NPCs as reported in original studies are retained as described, while the term NSPCs is used consistently throughout the review.

NSPCs are closely associated with neurological disorders. Dysfunction of NSPCs can promote the age-related advancement of neurodegeneration, exemplified by PD and AD, which present with exacerbated age-linked cognitive decline and significantly enhanced neuroinflammation [23]. Neural stem cell (NSC) transplantation reduced the level of the proinflammatory cytokines IL-6 and TNF-α and simultaneously attenuated inflammation-induced microgliosis and astrogliosis, thereby alleviating neuroinflammation [4]. NSC transplantation enhanced neuron and oligodendrocyte survival in the cerebral cortex and reduced neuronal apoptosis by inhibiting caspase-3 activity [4]. Subsequently, we describe how the transplantation of NSPCs improves the pathology of neurological disorders by inhibiting neuroinflammation, with a focus on the mechanisms underlying neuroinflammation, thereby providing novel therapeutic strategies for clinical treatment.

### 3.1. NSPCs and Alzheimer’s Disease

AD represents a neurodegenerative disorder characterized by progressive cognitive deterioration, gradual neuronal loss, and significant memory deficits [24]. Its two major pathological features are extracellular neuritic plaques derived from the deposition of β-amyloid (Aβ) protein and intracellular neurofibrillary tangles derived from the aggregation of hyperphosphorylated tau protein [25]. However, these two features cannot fully explain the entire pathological process of AD, implying the involvement of additional underlying mechanisms. Notably, studies have demonstrated sustained inflammatory responses in the brains of AD patients, which indicates that neuroinflammation acts as a key factor in the pathogenic process of AD [26].

Given the critical role of neuroinflammation in AD, NSC transplantation can attenuate neuroinflammation and improve spatial learning and long-term memory [27]. Specifically, NSC transplantation decreases IL-1, IL-6, and TNF-α in AD mice, reducing neuroinflammation by inhibiting glial cells and Toll-like receptor 4 (TLR4) [28]. Furthermore, NSCs may reduce caspase-1 and IL-1β levels in microglia and inhibit NLRP3 inflammasome expression by downregulating the p38α MAPK and GSK-3β signaling pathways, thereby further reducing neuroinflammation [29]. In addition to regulating neuroinflammation, transplanted NSCs can reduce Aβ deposition by upregulating the expression of Aβ-degrading enzymes [30] and decreasing tau hyperphosphorylation and amyloidogenesis [29], which directly targets the core pathological hallmarks of AD.

Notably, intranasal delivery of NSCs, a non-invasive transplantation approach, exhibits unique advantages. Intranasally transplanted NSCs can migrate into the brains of AD mice, primarily differentiating into neurons, including a proportion of cholinergic neurons, and alleviating synaptic loss. Moreover, intranasal transplantation upregulates vascular endothelial growth factor levels, promotes neurogenesis and neurovascular remodeling, and ultimately improves cognitive and cholinergic dysfunction [30]. Beyond these effects, NSC transplantation also downregulates autophagy, apoptosis, and pyroptosis in AD mice [31], drives microglial M2 polarization, and upregulates brain-derived neurotrophic factor (BDNF) and the synaptic protein PSD95, thereby promoting synaptic repair [31].

To enhance therapeutic effects, NSC transplantation with nanofiber scaffolds has been studied, showing enhanced neuroinflammation reduction by increasing p-Akt and IL-10 levels and decreasing cleaved caspase-3 and IL-1β [32]. In addition, this combined transplantation upregulates the expression of neurotrophic factors, including BDNF, ciliary neurotrophic factor, and IGF-1, promotes the recovery of synaptic function, and reduces Aβ levels, thereby significantly ameliorating cognitive dysfunction in AD rats [32].

Although NSPC transplantation can alleviate neuroinflammation and reduce Aβ deposition through multiple pathways, current studies are limited exclusively to animal models. Furthermore, these optimized delivery and combinatorial strategies possess distinct advantages yet show inconsistent therapeutic stability across preclinical investigations.

### 3.2. NSPCs and Parkinson’s Disease

PD is typified by the gradual degeneration of dopaminergic neurons and the intracellular aggregation of α-synuclein, which lead to severe motor complications [33]. Inflammatory dysregulation can occur concomitantly with these pathological changes, suggesting that neuroinflammation may play a role in the pathophysiology of PD [34,35].

Given the critical role of neuroinflammation in PD pathogenesis, transplantation of NSCs overexpressing nuclear receptor-related factor 1 (Nurr1) has been shown to ameliorate brain pathology in PD rats by attenuating neuroinflammation [36]. Nurr1 is an orphan nuclear receptor that interacts with cofactors to regulate the transcription of tyrosine hydroxylase (TH) and dopamine transporter, and is essential for dopamine synthesis [37]. In addition to its role in dopamine production, Nurr1 overexpression promotes the differentiation of NSCs into dopaminergic neurons, increases the number of TH-positive cells in the striatum, and reduces microglial activation, ultimately improving behavioral deficits in PD rats [36]. Furthermore, microglia overexpressing Nurr1 themselves display reduced levels of IL-1 and TNF-α, while also exhibiting elevated levels of BDNF and glial cell-derived neurotrophic factor (GDNF) [38]. This combined effect of enhanced dopaminergic differentiation and suppressed neuroinflammation acts synergistically to improve the hostile brain microenvironment, thereby producing long-term and significant beneficial effects in PD rats.

Beyond Nurr1-modified NSCs, other human NSC lines have also been explored for PD treatment. The hVM1 clone 32 cell line is a human NSC line that can differentiate in vitro to acquire dopaminergic neuronal characteristics. When transplanted into PD mice, these cells improved behavioral deficits [39]. However, the precise mechanism remains unclear.

NPCs also produce beneficial effects in PD. NPCs alleviate neuroinflammation, restore impaired motor function, and improve brain pathology in PD mice [40]. Specifically, NPCs decrease the expression of IL-1α, TNF-α, and IL-6, while increasing the level of IL-10 in PD mice [41]. They inhibit microglial activation and macrophage infiltration, and drive the phenotypic transition of microglia from the M1 to M2, thereby protecting substantia nigra neurons and ameliorating motor deficits in PD mice. These effects are attributed to the release of erythropoietin [41].

Similarly, transplantation of neural phenotype cells into PD rats can also improve motor deficits and protect dopaminergic neurons from loss [42]. The transplanted cells can survive and differentiate into various types of neurons, including TH-positive neurons; meanwhile, this transplantation can upregulate the expression of delta-like ligand 1 (DLL1) and the neurotrophic factor neurturin to optimize the local microenvironment. Additionally, the DLL1-Notch signaling pathway is involved in suppressing astrocyte and microglial activation, and in the neuroprotective process mediated by neural phenotype cells. These cells exert synergistic therapeutic effects through two mechanisms: inducing the secretion of neurotrophic factors and activating DLL1-Notch-mediated cell-contact signals [42]. Notably, these modified cell strategies exert protective effects through distinct molecular pathways but lack systematic comparison and rigorous validation for clinical translation. Furthermore, most relevant studies rely solely on rodent models, which cannot fully recapitulate the pathological progression of human PD.

### 3.3. NSPCs and Multiple Sclerosis

As a chronic immune-mediated condition of the CNS, MS presents core pathological hallmarks including inflammatory responses, demyelination, and progressive neurodegeneration. Its etiology remains incompletely understood, but it is considered to be multifactorial. Neuroinflammation is present at all stages of MS and is thought to play a vital role in the development of the disease [43,44].

To explore MS pathogenesis and potential therapies, experimental autoimmune encephalomyelitis (EAE) serves as the most widely used model [45]. Intraspinal injection of NSCs reduces neuroinflammation and promotes remyelination in EAE mice. Regulatory T cells (Tregs) accumulate at the NSC transplantation site, indicating that the recovery effect is associated with the emergence of Tregs [46]. A similar increase in Tregs is observed following NPC transplantation, which reduces demyelination and T-cell infiltration and alleviates neuroinflammation in EAE mice [47]. Tregs may participate in the maintenance of tissue homeostasis and promote tissue repair through interactions with NSPCs.

In addition to Treg-mediated immunomodulation, the inflammatory microenvironment and its molecular mechanisms have been further studied. Inflammatory stimulation activates inflammatory type 1 mononuclear phagocytes (IM1-MPs), leading to the accumulation of intracellular succinate and promoting IL-1β transcription. In addition, IM1-MPs can also release succinate into the extracellular space and upregulate the expression of its receptor SUCNR1, which further promotes IL-1β production through autocrine and paracrine mechanisms [48]. NSC transplantation in EAE mice reduces inflammation, lowers succinate levels, decreases immune cell infiltration, and drives microglial polarization toward an anti-inflammatory M2 phenotype, promoting anti-inflammatory effects [49]. Mechanistically, succinate secreted by IM1-MPs activates SUCNR1 on NSCs, causing the release of prostaglandin E2 and the removal of extracellular succinate, thereby significantly reducing IL-1β levels and alleviating axonal loss and demyelination [49].

Optimizing stem cell delivery is another key to enhancing NSC function and therapeutic efficacy in MS [50]. E-selectin ligands are specific carbohydrate structures expressed on the cell surface that can bind to E-selectin on vascular endothelium, mediating cell rolling, adhesion, and transvascular migration. They serve as a key molecular basis for stem cells to efficiently enter lesions in the CNS [51]. Following intravenous administration, this glycoengineering strategy ameliorates the clinical course of EAE mice, as evidenced by reduced inflammation and improved oligodendrocyte and axonal integrity [50].

NPCs can therapeutically benefit EAE through unique mechanisms. By secreting TGF-β2, NPC transplantation significantly modulates the immune system, inhibiting inflammatory monocyte-derived cell activation and encouraging their shift to an anti-inflammatory state [52]. In addition, NPCs reduce the production of granulocyte-macrophage colony-stimulating factor (GM-CSF) and decrease the levels of IL-1β, TNF-α, and IL-23, thereby inhibiting the terminal differentiation of myelin-reactive pathogenic T helper cells. Reducing GM-CSF, alongside TGF-β2 from NPCs, alleviates neuroinflammation, aiding neurological recovery and improving motor function in EAE mice [52]. Collectively, various NSPC-based therapies exert protective effects through distinct immune-regulatory pathways in EAE models. Nevertheless, the EAE model cannot fully recapitulate the complex relapsing and progressive course of human MS. In addition, NSPCs from different sources exhibit highly variable therapeutic efficacy, and standardized evaluation criteria are still lacking, resulting in poorly defined individualized clinical regimens that hinder further translational development.

### 3.4. NSPCs and Ischaemic Stroke

Ischemic stroke is characterized by arterial occlusion caused by embolism or thrombosis, which leads to metabolic insufficiency in the CNS and ultimately results in brain tissue damage and cell death [53]. Among these pathophysiological processes, neuroinflammation has emerged as a core contributor to the pathophysiological processes of stroke [54,55]. NSC transplantation exerts beneficial effects against neuroinflammation; specifically, it can reduce infarct volume, blunt the synthesis and release of TNF-α, IL-6, and IL-1β, and simultaneously downregulate the expression of matrix metalloproteinase (MMP)-3 and MMP-9 [56]. It reduces pyroptosis and necroptosis in astrocytes and inhibits necroptosis in neurons, thereby improving functional outcomes in middle cerebral artery occlusion (MCAO) mice [57].

Similarly, NPCs can also reduce microglial activation and promote the transition to the M2 microglial phenotype, thereby alleviating neuroinflammation. Meanwhile, they promote neurogenesis in the subventricular zone and enhance angiogenesis. Transplanted cells can form synaptic connections with host striatal neurons, thereby establishing neural networks between the grafted cells and the host brain [58]. These effects may be associated with elevated phosphorylation of Akt and ERK, which in turn increase phosphorylation of their downstream transcription factor CREB [59]. In this process, GSK3β normally inhibits CREB phosphorylation, but is inactivated upon its own phosphorylation. Increased phosphorylated GSK3β is observed following NPC transplantation, further promoting CREB activation. Thus, the synergistic effect between enhanced CREB phosphorylation and relieved GSK3β-mediated inhibition may underlie the promotion of neurogenesis [59]. However, it is worth noting that NPC transplantation increased the incidence and area of hemorrhage in the ischemic hemisphere and induced pro-inflammatory responses by elevating leukocyte infiltration in the peripheral blood of hyperlipidemic MCAO mice [60].

To further improve the therapeutic outcome of NPC transplantation in ischemic stroke, combined transplantation strategies have been developed and validated. Combined transplantation of NPCs with a nutritional hydrogel produces superior anti-neuroinflammatory effects. This strategy enhanced stroke recovery and ameliorated behavioral deficits in MCAO mice [61].

Besides nutritional hydrogels, other approaches can boost the therapeutic effects of NPCs. For example, chondroitin sulfate (CS-A) has been shown to improve cellular and trophic factor-mediated repair after brain injury. Treatment with CS-A-encapsulated NPCs (CS-A + NPCs) promotes vascular remodeling and regeneration, increases regional cerebral blood flow, and ameliorates motor deficits [62]. CS-A + NPC transplantation upregulates the levels of IL-10 and downregulates macrophage inflammatory protein 1a and 1b, as well as IL-4 and IL-6 [63]. These effects may be attributed to the ability of CS-A to upregulate the expression of monocyte chemoattractant protein 1 and basic fibroblast growth factor, thereby enhancing NPC-mediated repair [62,63].

Apart from optimizing transplantation strategies, advances in NPC preparation methods have also laid a foundation for better clinical translation. Fu Z et al. established an efficient and reproducible suspension culture platform that enables the direct generation of NPCs from human induced pluripotent stem cells (iPSCs) without passaging before cell collection. This 3D aggregate-based NPC differentiation method can effectively facilitate clinical translation. The NPCs generated using this approach significantly improved motor dysfunction and brain atrophy in MCAO mice, and were able to migrate to the ischemic region and differentiate into multiple neuronal types. Transplantation of these NPCs effectively increased the number of M2 microglia and reduced neuroinflammation [64]. Notably, divergent therapeutic outcomes exist across different transplantation strategies, and the underlying regulatory mechanisms have not been fully elucidated. Moreover, the safety risks such as elevated hemorrhagic incidence in specific disease backgrounds further restrict the clinical translation of these stem cell therapies.

### 3.5. NSPCs and Intracerebral Hemorrhage

Intracerebral hemorrhage (ICH) is widely recognized as one of the most devastating subtypes of stroke, primarily causing physical brain damage due to the rapid formation of hematoma and mass effect [65]. Neuroinflammation plays a key role in secondary brain injury post-ICH, and reducing inflammation can significantly mitigate ICH-induced brain damage [66].

NSC transplantation can alleviate ICH-induced neurological deficits by suppressing neuroinflammation. Specifically, NSCs can drive the phenotypic switch of activated microglia from M1 to M2, while inhibiting the production of IL-6, IL-1β, and IL-18 [67]. In addition, NSC transplantation downregulates the expression of inflammatory markers associated with canonical pyroptosis, including active NF-κB, NLRP3 inflammasome, and active caspase-1. These findings indicate that NSC transplantation alleviates post-ICH neuroinflammation and exerts neuroprotective effects mainly by blocking the NF-κB-mediated pyroptosis pathway [67].

Therefore, targeting neuroinflammation and pyroptosis through NSC transplantation may be recognized as a promising therapeutic strategy for ICH. However, current research remains insufficient to provide adequate safety and efficacy data for clinical translation, warranting further investigation into additional potential therapeutic targets and signaling pathways.

### 3.6. NSPCs and Spinal Cord Injury

Spinal cord injury (SCI) is a devastating neurological disorder that causes severe sensorimotor dysfunction. The initial SCI disrupts the integrity of the blood–spinal cord barrier, which in turn triggers secondary injuries such as neuroinflammation, neuronal loss, and apoptosis [68,69]. Inflammation following SCI is complex and orchestrated by multiple cell types and various inflammatory cytokines. Although inflammation after SCI exerts several beneficial effects, extensive infiltration of immune cells is a major contributor to neurodegeneration [70]. Therefore, in therapeutic strategies for SCI, alleviating neuroinflammation is crucial for promoting neurological functional recovery.

NSC transplantation is an important strategy for alleviating neuroinflammation after SCI, especially when combined with other agents or cells to achieve synergistic effects. A typical example is the co-transplantation of NSCs with composite hydrogels. The composite hydrogel can scavenge reactive oxygen species to protect NSCs from oxidative damage. At the same time, microglial polarization toward the M2 phenotype is significantly enhanced by NSCs, thereby reducing neuroinflammation and inhibiting glial scar formation [71]. In addition, the composite hydrogel can release nitric oxide, thereby inducing calcium influx and upregulating the cAMP-PKA pathway, which drives NSCs to differentiate into neurons and integrate with the host neural circuits, thus replenishing damaged neurons [71]. Co-transplantation of NSCs with other cell types also shows favorable synergistic effects. The combined transplantation of NSCs and bone marrow mesenchymal stem cells (BMSCs) attenuates the secretion of IL-6, IL-1, and TNF-α, reduces the inflammatory level after SCI, and alleviates damage to neural cells; meanwhile, it increases the expression of nerve growth factor (NGF) and BDNF [72]. Co-transplantation of NSCs with activated olfactory ensheathing cells (aOECs) also improves neurological function in rats with SCI [73]. aOECs can significantly enhance the neuronal differentiation and migration of NSCs, which may be related to the activation of the Wnt3/β-catenin pathway [73].

NPC transplantation can also inhibit the inflammatory response after SCI and restore hindlimb motor function in SCI rats [74]. Transcriptome analysis reveals that this is mainly achieved by upregulating genes associated with myelination and oligodendrocytes, while downregulating genes involved in cytokine production and immune system responses [74]. However, the persistent local inflammatory microenvironment and newly formed glial scar constitute a formidable physical barrier post-SCI, impeding the colonization of transplanted NSPCs at the lesion site and their differentiation into functional neural cells. Accordingly, the long-term survival and integration of transplanted NSPCs within this hostile inflammatory microenvironment still demand further optimization.

### 3.7. NSPCs and Traumatic Brain Injury

Traumatic brain injury (TBI), defined as brain parenchymal damage resulting from external mechanical insults, is a leading cause of global mortality and long-term acquired disability. Primary mechanical injury triggers deleterious biochemical cascades, which further lead to secondary injury. Among these, neuroinflammation is a key factor, and the extensive and persistent damage it causes prolongs the clinical course and worsens the prognosis of TBI [75,76].

NSC transplantation can inhibit TBI-induced white matter neuroinflammation, thereby improving diffuse white matter pathology [77]. It also reduces astrogliosis and enhances neurogenesis by promoting the proliferation and differentiation of endogenous NPCs [78]. This may be attributed to the fact that a subset of transplanted NSCs express the Sonic hedgehog (Shh) ligand. Shh signaling can modulate neuroinflammation and stimulate neuroregeneration in the subventricular zone, thereby alleviating the pathological damage caused by TBI [77]. Nevertheless, further systematic investigations are required to elucidate the molecular mechanisms in detail and to identify more effective therapeutic targets to facilitate clinical translation.

### 3.8. NSPCs and Closed Head Injury

Closed head injury (CHI) is typically caused by mechanical impact and leads to widespread diffuse damage [79]. CHI can exacerbate neurodegeneration and induce excessive microglial activation, and even result in cognitive deficits and learning impairments [80]. Neuroinflammation contributes critically to this process, and dysregulated neuroinflammation constitutes an important secondary injury mechanism in CHI, which is often associated with adverse outcomes [81].

NSC transplantation can reduce immune cell infiltration and downregulate the expression of TNF-α, IL-1β, and IL-8, thereby alleviating neuroinflammation [82]. In addition, NSC transplantation upregulates BDNF, GDNF, and IGF-1, increases the population of surviving neurons, and ultimately improves neurological function in CHI mice [82,83]. The complement system is involved in these effects. As an essential part of the immune system, the complement system mediates neuroinflammation and promotes neuronal apoptosis after CHI. Transplanted NSCs express complement receptor 1-related protein y (Crry), which can inhibit complement activation and thereby prevent persistent neuroinflammation and neuronal damage [84]. Furthermore, CXCL12 secreted by activated microglia after CHI can activate Akt in NSCs. Akt activation further enhances the activities of CXCL12/CXCR4 and Crry in NSCs, and reduces the formation of complement C5b-9 complexes [83]. On the other hand, TNF-α produced by activated microglia can bind to TNFR1 on the surface of NSCs, and the TNF-α/TNFR1 signaling pathway positively regulates NF-κB and CXCR4 in NSCs [85].

**Figure 1:**
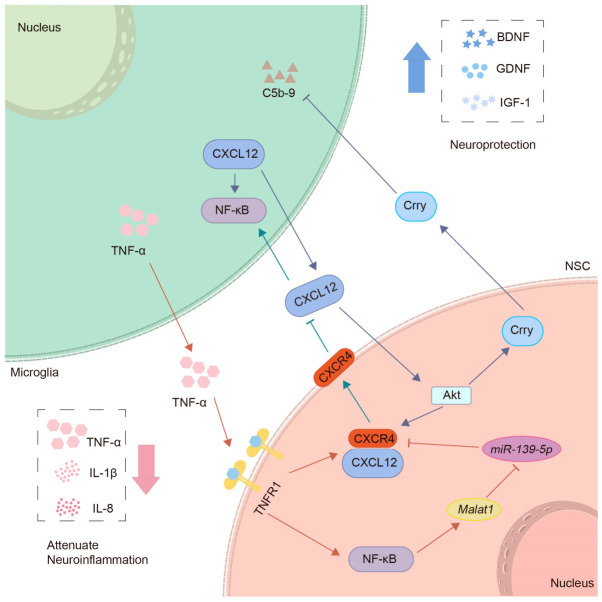
**Mechanistic diagram of NSCs-mediated regulation of microglial activation and neuroinflammation after CHI.** After CHI, activated microglia secrete CXCL12 and TNF-α. CXCL12 activates Akt in NSCs to upregulate Crry and CXCR4, and Crry inhibits C5b-9 formation. TNF-α binds to TNFR1 on NSCs and promotes NF-κB and CXCR4 activation, which upregulates *Malat1*. *Malat1* functions as a molecular sponge for *miR-139-5p* to further increase CXCR4 expression. Increased CXCR4 neutralizes microglial CXCL12, thereby blocking NF-κB activation in microglia. This cascade reduces pro-inflammatory cytokines, including IL-1β, IL-8, and TNF-α, elevates BDNF, GDNF, and IGF-1, and ultimately improves neurological function after CHI. CHI, Closed head injury; TNF-α, Tumor necrosis factor-α; NSC, Neural stem cell; Crry, Complement receptor 1-related protein y; C5b-9, Complement component 5b-9; TNFR1, Tumor necrosis factor receptor 1; NF-κB, Nuclear factor-kappa B; IL, Interleukin; BDNF, Brain-derived neurotrophic factor; GDNF, Glial cell line-derived neurotrophic factor; IGF-1, Insulin-like growth factor 1.

Further mechanistic studies reveal that *Malat1* is the most significantly upregulated long non-coding RNA (lncRNA) in transplanted NSCs [85]. lncRNAs can act as competing endogenous RNAs (ceRNAs) of microRNAs to modulate target mRNA expression, thereby playing key regulatory roles in neuroinflammation [86]. *Malat1* can regulate CXCR4 expression by sponging miR-139-5p. Specifically, NF-κB activation in NSCs can upregulate *Malat1* expression, and *Malat1*, conversely, acts as a ceRNA for miR-139-5p, thereby activating CXCR4 in NSCs. In addition, CXCR4, located on NSCs’ membranes, can neutralize CXCL12 released from activated microglia, thereby blocking the effect of CXCL12/CXCR4 signaling in mediating NF-κB activation in microglia [85]. Therefore, the crosstalk among the above signaling pathways may form a regulatory network that modulates the interaction between NSCs and microglia (Figure 1), thereby alleviating neuroinflammation and improving the pathological condition of CHI. However, the pathological severity of CHI models is greatly influenced by factors such as impact force and injury location. The CHI models used in current studies are poorly standardized, resulting in poor comparability of experimental results. Furthermore, the long-term effect of NSPC transplantation on blood–brain barrier repair following injury remains unclear, and numerous technical bottlenecks still hinder clinical translation.

### 3.9. NSPCs and Posthemorrhagic Hydrocephalus

Post-hemorrhagic hydrocephalus (PHH) is a severe complication that occurs in preterm infants associated with intraventricular hemorrhage (IVH). PHH is closely associated with neuroinflammatory responses induced by the degradation of blood-derived products [87,88]. Therefore, modulating neuroinflammation may ameliorate the pathology of PHH.

NSC transplantation alleviates neuroinflammation and improves neurological dysfunction after PHH [89]. However, Sox2-overexpressing NSCs (NSCs-Sox2) exhibit superior efficacy compared with unmodified NSCs. NSCs-Sox2 transplantation decreased TNF-α and IL-6 levels and enhanced M2 polarization of microglia, and upregulated the expression of BDNF and NGF. NSCs-Sox2 transplantation also significantly reduced ventricular volume in IVH mice, improved cognitive functions, and promoted neuronal regeneration and angiogenesis. These effects may be attributed to NSCs-Sox2-mediated inhibition of the IVH-activated TLR4 inflammatory pathway in the mouse choroid plexus [89]. These results suggest that NSC-Sox2 transplantation may be a promising therapeutic approach for PHH by suppressing neuroinflammation and improving neurological function. However, the safety of Sox2-modified NSCs has not been fully established, and their long-term regulatory effects on cerebrospinal fluid circulation after transplantation remain unclear. Relevant preclinical research data are also extremely scarce. Therefore, further research is necessary to validate the efficacy and safety of this strategy prior to clinical translation.

### 3.10. NSPCs and Autism Spectrum Disorder

Autism spectrum disorder (ASD) encompasses a heterogeneous group of neurodevelopmental disorders, characterized by core clinical features including persistent deficits in social interaction and communication skills. Restricted and repetitive behaviors often accompany these deficits. Notably, neuroinflammation is implicated in the pathogenesis of ASD [90,91]. Preventing and targeting neuroinflammation in ASD may contribute to reducing the incidence of ASD in fetuses and children.

NSC transplantation can reduce the levels of IL-1β, IL-6, and TNF-α, inhibit microglial activation, and upregulate IL-10 expression, thereby alleviating neuroinflammation in ASD mice [92]. In addition, NSC transplantation can restore gut microbiota homeostasis and ultimately improve core behavioral deficits in ASD mice, including enhanced social interaction and decreased repetitive behavior [92]. However, the application of NSC transplantation in ASD via alleviating neuroinflammation remains limited. In addition, the long-term efficacy, safety, and ethical considerations of cell transplantation in improving core symptoms of ASD have not been systematically evaluated, and clinical translation remains at the exploratory stage. Further studies are warranted to elucidate the underlying mechanisms of NSC transplantation in the treatment of ASD.

### 3.11. Common Mechanisms and Source Heterogeneity of NSPCs Across Disease Models

NSPCs exhibit significant heterogeneity in their sources, including embryonic-derived, adult tissue-derived, and iPSC-derived subpopulations. They also encompass human NSCs and induced NSCs obtained through reprogramming. In addition, genetically modified NSPC variants have been developed for targeted regulation of neuroinflammation (Table 1). Although all NSPCs exert anti-inflammatory effects in neurological disease models, their biological properties differ substantially across sources, leading to distinct therapeutic efficacy, molecular mechanisms, and translational potential in neuroinflammation control. Their core similarities and source-specific differences are summarized below.

**Figure 2:**
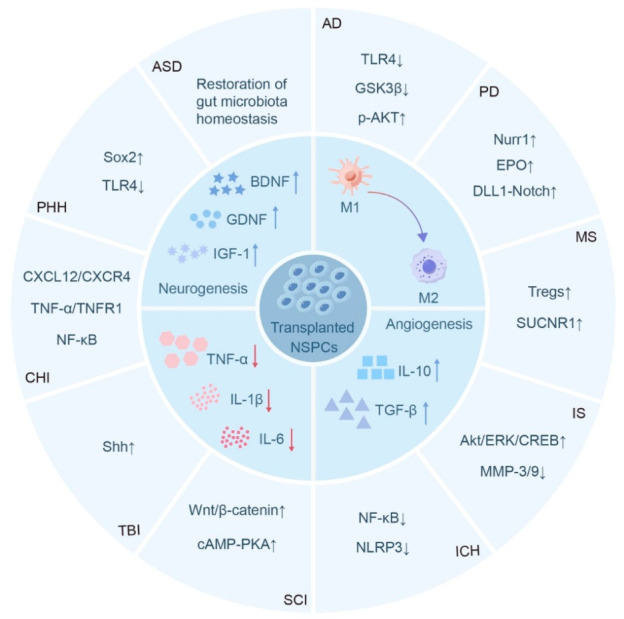
**Mechanisms of transplanted NSPCs in regulating neuroinflammation across neurological disorders.** Transplanted NSPCs exert multifaceted anti-inflammatory and neuroprotective effects in various neurological diseases, acting through both shared and disease-specific pathways. Common therapeutic mechanisms include: promoting microglial polarization from the pro-inflammatory M1 to anti-inflammatory M2 phenotype; enhancing the secretion of neurotrophic factors (BDNF, GDNF, IGF-1); increasing anti-inflammatory cytokines (IL-10, TGF-β); reducing pro-inflammatory cytokines (TNF-α, IL-1β, IL-6); and supporting angiogenesis and neurogenesis. Disease-specific regulatory pathways are illustrated for major neurological disorders, including Alzheimer’s disease (AD), Parkinson’s disease (PD), multiple sclerosis (MS), ischemic stroke (IS), intracerebral hemorrhage (ICH), spinal cord injury (SCI), traumatic brain injury (TBI), closed head injury (CHI), post-hemorrhagic hydrocephalus (PHH), and autism spectrum disorder (ASD). BDNF, Brain-derived neurotrophic factor; cAMP-PKA, Cyclic adenosine monophosphate-protein kinase A; DLL1, Delta-like ligand; EPO, Erythropoietin; GDNF, Glial cell line-derived neurotrophic factor; GSK3β, Glycogen synthase kinase 3β; IGF-1, Insulin-like growth factor 1; IL, Interleukin; MMP, Matrix metalloproteinase; NF-κB, Nuclear factor-κB; NLRP3, NOD-like receptor pyrin domain-containing protein 3; Notch, Notch receptor; Nurr1, Nuclear receptor-related factor 1; Shh, Sonic hedgehog; SUCNR1, Succinate receptor; TGF-β, Transforming growth factor-β; TLR4, Toll-like receptor 4; TNF-α, Tumor necrosis factor-α; TNFR1, Tumor necrosis factor receptor 1; Wnt/β-catenin, Wingless-related integration site/β-catenin; CXCL12/CXCR4, C-X-C motif chemokine ligand 12/C-X-C motif chemokine receptor 4.

A core set of conserved mechanisms underlies the anti-inflammatory and therapeutic effects of NSPC transplantation across neurological disorders. First, all functional NSPC subpopulations exert anti-inflammatory effects predominantly via paracrine regulation rather than direct cell replacement (Figure 2). They upregulate anti-inflammatory cytokines (IL-10, TGF-β) and promote the secretion of neurotrophic factors (BDNF, GDNF, IGF-1), together with a variety of regulatory molecules. These actions suppress microglial pro-inflammatory activation and reactive astrogliosis, disrupt the pro-inflammatory positive feedback loop among glial cells, and preserve BBB integrity. Second, NSPCs remodel the CNS immune microenvironment by promoting microglial polarization toward an anti-inflammatory phenotype, reducing the secretion of pro-inflammatory cytokines including IL-1β, IL-6 and TNF-α, and inhibiting infiltration of peripheral immune cells into lesions. Third, NSPCs universally integrate anti-inflammatory action with neural repair functions. While alleviating excessive neuroinflammation, they promote endogenous neurogenesis, oligodendrocyte regeneration and angiogenesis, thereby reconstructing the damaged CNS microenvironment and restoring neurological function.

Source-specific properties strongly influence the efficacy and safety of NSPC-mediated neuroinflammation regulation. Embryonic-derived NSPCs exhibit exceptional proliferative capacity and multilineage differentiation potential, enabling efficient modulation of neuroinflammation in AD [28], PD [40], TBI [78], and ischemic stroke [57,59]. Mechanistically, they upregulate AKT/ERK/CREB signaling [59], inhibit overactivation of microglial TLR4 [28], block inflammatory cascade amplification, and regulate pyroptosis and apoptosis pathways [57] to reduce secondary neural injury.

In contrast, adult tissue-derived NSPCs exert anti-inflammatory effects in MS [52], SCI [74], and TBI [77]. The anti-inflammatory effect of these cells is mainly achieved by reducing the secretion of GM-CSF [52] and regulating the Shh signaling pathway [77], thereby alleviating neuroinflammation and reducing neuronal damage.

iPSC-derived NSPCs are a type of cells that can be obtained on a large scale and have autologous origin characteristics. They exhibit significant anti-inflammatory effects in MS [47], ischemic stroke [58], and SCI [72], and can exert anti-inflammatory effects by increasing Tregs [47].

Human NSPCs exert anti-inflammatory effects in AD [27,30,32], PD [39], MS [46,49], ischemic stroke [56], and ICH [67] by activating IM1-MPs [49], elevating p-AKT [32], elevating Tregs [46], downregulating MMP3/9 [56] and blocking the NF-κB-mediated pyroptosis pathway [67].

Induced NSCs have already been discussed in the section on NSPCs and ICH, and will not be elaborated upon here.

Beyond naturally derived NSPCs, genetically engineered NSPC variants, including NSCs-Nurr1 [36], NSCs-Sox2 [89], and 3KO-NSCs [92] enable targeted CNS inflammation regulation. In PD models, NSCs-Nurr1 can not only inhibit pro-inflammatory cytokines, but also promote the differentiation of dopaminergic neurons [36]. In PHH models, NSCs-Sox2 inhibit the TLR4 signaling pathway and thereby promote the polarization of microglia toward an anti-inflammatory phenotype [89]. In addition, in ASD models, 3KO-NSCs suppress the expression of pro-inflammatory factors while upregulating the production of anti-inflammatory factors [92].

In summary, selecting the sources of NSPCs for neuroinflammation therapy requires comprehensive balancing of therapeutic efficacy, safety, and clinical feasibility according to disease-specific characteristics.

## 4. Discussion

### 4.1. Key Barriers to Clinical Translation of NSPC-Based Therapy

Despite the promising preclinical efficacy of NSPC transplantation in modulating neuroinflammation and alleviating neurological disease pathology, clinical translation remains hindered by multiple unresolved bottlenecks, including cell source standardization, safety, delivery strategies, and microenvironmental adaptability.

A fundamental barrier is the lack of standardized NSPC sources and culture protocols. Currently, NSPC isolation, induction, and expansion lack unified industry standards: embryonic-derived NSCs have inconsistent differentiation potential across different donors, adult-derived NSPCs have low extraction efficiency and batch-to-batch variation, and iPSC-derived NPCs exhibit heterogeneous differentiation due to different reprogramming and induction methods [93,94]. In addition, the absence of standardized quality control leads to poor reproducibility of therapeutic effects in preclinical studies, which cannot meet the requirements of clinical trial standardization [95].

Unresolved safety risks including tumorigenicity, immunogenicity, and off-target effects restrict clinical application. Embryonic-derived NSPCs are associated with ethical concerns, and both these cells and unpurified NSPC populations carry a tumorigenic risk due to residual undifferentiated cells [96,97]. Allogeneic NSPC transplantation triggers adaptive immune rejection. This immune response not only impairs the survival and integration of transplanted cells into the host neural tissue but also limits the long-term therapeutic efficacy of cell-based strategies for neurological disorders. Even autologous iPSC-derived NPCs may induce innate immune responses due to epigenetic modifications during reprogramming. Moreover, immune suppression regimens, while commonly used to alleviate rejection, carry potential risks of systemic infection, further complicating the clinical translation of NSPC transplantation [96,98]. Genetically modified NSPCs face risks of off-target genetic editing and abnormal gene expression [99], which may alter their anti-inflammatory and differentiation properties and cause unpredictable pathological effects. Additionally, adult tissue-derived NSPCs have limited proliferation and differentiation capacity, which restricts their therapeutic efficacy in acute severe CNS injuries.

Inefficient delivery strategies compromise NSPC survival and targeting efficiency in the CNS. Invasive intracerebral or intraspinal transplantation enables direct engraftment but carries risks of surgical trauma, hemorrhage, and glial scar formation at the injection site. Systemic intravenous infusion results in extensive peripheral entrapment, with few cells crossing the BBB to reach the injured CNS region. Non-invasive routes such as intranasal delivery show translational potential but are limited by low NSPC survival and migration efficiency [100,101]. In addition, the optimal transplantation dosage and timing remain undefined: acute transplantation is hampered by severe post-injury inflammation with high TNF-α and IL-1β that induce NSPC apoptosis, whereas delayed transplantation fails to suppress early neuroinflammatory progression [102].

Transplanted NSPCs also display poor adaptability to the pathological CNS microenvironment, resulting in low graft survival and weakened anti-inflammatory effects. The injured niche is characterized by excessive neuroinflammation, oxidative stress, and glial scarring, which collectively promote NSPC apoptosis and suppress proliferation and differentiation [103]. The glial scar further blocks NSPC migration and integration into host neural circuits [104]. In addition, host comorbidities such as hyperlipidemia can exacerbate adverse outcomes, as seen in increased intracerebral hemorrhage and pro-inflammatory leukocyte infiltration in hyperlipidemic stroke models [60].

Finally, reliable preclinical efficacy and safety evaluation systems are lacking. Most studies rely on rodent models that poorly recapitulate complex human neuropathology, and the scarcity of clinically relevant large animal models limits accurate prediction of therapeutic and safety profiles in humans.

### 4.2. Adjuvant Strategies to Optimize NSPC-Mediated Neuroinflammation Modulation

To overcome the above clinical translation barriers and enhance the anti-inflammatory and neurorepair efficacy of NSPC transplantation, several adjuvant strategies have been established and validated in preclinical studies, which mainly include biomaterial combination, genetic modification, small molecule coupling, and co-transplantation with other cell types. These strategies synergize with NSPCs to improve their survival, targeting, and anti-inflammatory capacities, and form a multi-modal therapeutic system for neuroinflammation-related neurological diseases.

Co-transplantation of NSPCs with biomaterials/scaffolds optimizes the graft microenvironment and enhances anti-inflammatory efficacy. These materials scavenge reactive oxygen species to protect NSPCs from oxidative damage, inhibit glial scar formation and promote the migration and integration of NSPCs. In AD models, NSPC transplantation combined with peptide nanofibers upregulates the expression levels of p-Akt and IL-10, which markedly enhances the inhibition of neuroinflammation and improves cognitive function [32]. In SCI models, composite hydrogels synergize with NSPCs via the cAMP-PKA pathway to promote neuronal differentiation and anti-inflammatory microglial polarization [71]. In ischemic stroke models, the co-delivery of nutritional hydrogels with NSPCs also achieves prominent anti-inflammatory effects [61].

Genetic modification enables targeted improvement of NSPC-mediated neuroinflammation regulation and directed differentiation. Genetic engineering of NSPCs to overexpress key anti-inflammatory and neurotrophic genes significantly improves their therapeutic efficacy in disease-specific models: NSCs-Nurr1 suppresses pro-inflammatory cytokines and supports dopaminergic neuron regeneration in PD [36]; NSCs-Sox2 inhibits the TLR4 pathway and promotes anti-inflammatory microglial polarization in PHH models [89]. In addition, glycoengineered NSPCs expressing E-selectin ligands show enhanced BBB penetration and targeted lesion accumulation, significantly reducing neuroinflammation and demyelination in MS [50].

Small molecule conjugation enhances NSPC survival and paracrine activity. For example, CS-A microencapsulation upregulates monocyte chemoattractant protein 1 and basic fibroblast growth factor expression, promoting vascular remodeling and neuroinflammation inhibition in ischemic stroke [62,63].

NSPC co-transplantation with other cell types exerts synergistic anti-inflammatory and neurorepair effects. In SCI, co-transplantation with BMSCs reduces IL-6, IL-1, and TNF-α, and upregulates NGF and BDNF expression, where BMSCs optimize the inflammatory microenvironment for NSPC survival while NSPCs promote neuronal regeneration [72]. Co-transplantation with aOECs enhances neuronal differentiation and migration of NSPCs by activating the Wnt3/β-catenin pathway, improving hindlimb motor function recovery [73].

These adjuvant strategies effectively compensate for the intrinsic limitations of NSPCs, including low survival rate, poor targeting, and attenuated anti-inflammatory function in the pathological microenvironment, and provide a feasible approach for the clinical translation. Combinatorial multimodal strategies represent a key direction for advancing NSPC-based therapies targeting neuroinflammation.

### 4.3. Future Research Directions

Although combination therapy has shown promising progress, critical challenges remain to be addressed, including targeted cell delivery, sustained regulation of the inflammatory microenvironment, and rigorous clinical safety evaluation.

The crosstalk among transplanted NSPCs, host resident cells, and microenvironmental signals represents a key determinant for successful clinical translation of neural transplantation. Specific immune depletion of immune cell subsets can regulate the therapeutic efficacy of transplanted cell populations [105]. Therefore, future studies are required to accurately evaluate the interaction between transplanted cells and host tissues to delineate, in greater detail, the cellular and molecular events accompanying NSPC transplantation.

Given the wide variety of NSPC sources and types, the feasibility of large-scale production has become one of the key factors hindering their clinical translation [106]. It is of great significance to establish stable and efficient in vitro culture and expansion protocols to achieve standardized mass production of NSPCs while ensuring cell viability, safety, and biological functions, thereby improving their clinical translation potential and expanding their clinical application scope. The establishment of well-defined cultural protocols and stringent quality control could help achieve this goal. In addition, the involvement of large enterprises may allow for the production of large quantities of cells through standardized industrial manufacturing processes, further accelerating translation into clinical trials.

The pathogenesis of many neurological diseases is extremely complex and not yet fully elucidated. Their occurrence and progression often involve the combined participation of multiple factors and pathways, covering genetic susceptibility, abnormal cellular metabolism, synaptic dysfunction, oxidative stress-induced injury, immune imbalance, and other aspects. Among numerous pathological processes, neuroinflammation is only one key link and an important component of the complex pathological network. Analyzing its crosstalk with other pathways and exploring other pathways will provide a key theoretical reference for clarifying disease pathogenesis.

In the future, it is necessary to establish unified culture and quality control standards for NSPCs, develop more targeted delivery systems, and conduct long-term safety studies in large animal models, so as to provide a solid foundation for clinical trials.

## 5. Conclusions

This review mainly summarizes the progress over the past decade regarding how NSPCs improve the pathology of neurological diseases by regulating neuroinflammation-related mechanisms. We conclude that NSPC-based therapeutic strategies exhibit considerable potential for the development of treatments against neurological diseases. In conclusion, NSPC transplantation holds great promise as a potential therapeutic strategy for neurological disorders. Nevertheless, practical clinical challenges need to be addressed, and translational research data should be interpreted in more depth to maximize the clinical value of NSPC-based therapy in the treatment of neurological diseases.

## Figures and Tables

**Table 1:** Summary of NSPCs’ application and mechanism in nervous system diseases.

Diseases	Experimental Model	NSPCs Source	Delivery Routes	Outcome	Mechanistic Pathways	References
**AD**	APP/PS1 mice/5XFAD mice	Human NSCs, embryonic-derived NSCs, NE4C cells	Intracerebral injection, intranasal delivery	Improvement of cognitive function, reduced Aβ deposition	Inhibition of the TLR4/NLRP3 signaling pathway, downregulation of p38α MAPK and GSK-3β signaling pathways	[27,28,29,30,31,32]
**PD**	PD rats/mice	Embryonic-derived NSCs/NPCs, human NSCs, NSCs-Nurr1, neural phenotype cells	Intracerebral injection, intravenous injection	Improvement of motor function, increases in the number of TH-positive cells	Upregulate the DLL1-Notch signaling pathway	[36,39,40,41,42]
**MS**	EAE/JHMV-infected mice	Human NSCs, iPSC-derived NSCs, adult NSCs	T9 thoracic vertebral injection, intravenous injection, intracerebral injection, intrathecal injection	Neurological recovery, improvement of motor function, attenuation of axonal loss and demyelination	Increase in Tregs, activation of SUCNR1 on NSCs	[46,47,49,50,52]
**Ischemic stroke**	MCAO mice/rats	Human NSCs, embryonic-derived NSCs, iPSC-derived NPCs, hESC-derived NPCs	Intracerebral injection	Reduction in infarct volume, recovery of motor function	Inhibition of MMP-3/9, activation of the Akt/ERK/CREB signaling pathway	[56,57,58,59]
**ICH**	ICH rats	Placenta hMSC-derived NSCs	Intracerebral injection	Improvement of neurological deficits	Inhibition of NF-κB/NLRP3	[67]
**SCI**	SCI mice/rats	Adult NSCs, hiPSC-derived NSCs	Lesioned spinal cord	Recovery of hindlimb motor function, reduction in glial scar formation	Upregulation of the cAMP-PKA pathway, activation of the Wnt3/β-catenin pathway	[71,72,74]
**TBI**	TBI mice	Adult NSCs, embryonic-derived NSCs	Intracerebral injection	Improving diffuse white matter pathology, reduces astrogliosis	NSCs express Shh ligand	[77,78]
**CHI**	CHI mice	iNSCs	Intracerebral injection	Improvement of neurological function, increase in neuronal survival	TNF-α/TNFR1, NF-κB, Malat1/miR-139-5p/Cxcr4 and CXCL12/CXCR4 form a regulatory network	[82,83,84,85]
**PHH**	PHH rats	NSCs-Sox2	Intracerebral injection	Reduction in ventricular volume, improvement of cognitive function, promotion of neuronal regeneration and angiogenesis	Inhibition of the TLR4 signaling pathway	[89]
**ASD**	VPA-induced ASD mice	3KO-hiPSC-NSCs	Combined intravenous and intracerebral administration	Improvement of social behaviors, reduction in repetitive behaviors	Inhibition of pro-inflammatory cytokines, regulation of gut microbiota	[92]

Abbreviations: AD, Alzheimer’s disease; ASD, Autism spectrum disorder; Aβ, β-amyloid; CHI, Closed head injury; DLL1, Delta-like ligand 1; EAE, Experimental autoimmune encephalomyelitis; ICH, Intracerebral hemorrhage; MCAO, Middle cerebral artery occlusion; MMP, Matrix metalloproteinase; MS, Multiple sclerosis; NPCs, Neural progenitor cells; NSCs, Neural stem cells; NSCs-Sox2, Sox2-overexpressing NSCs; NSCs-Nurr1, Nurr1-overexpressing NSCs; NSPCs, Neural stem/progenitor cells; Nurr1, Nuclear receptor-related factor 1; PD, Parkinson’s disease; PHH, Post-hemorrhagic hydrocephalus; SCI, Spinal cord injury; Shh, Sonic hedgehog; TBI, Traumatic brain injury; TH, Tyrosine hydroxylase; TLR4, Toll-like receptor 4; TNF-α, Tumor necrosis factor-α; Tregs, Regulatory T cells; iPSC, induced pluripotent stem cell; hiPSC, human induced pluripotent stem cell; hMSC, human mesenchymal stem cell; hESC, human embryonic stem cell; iNSCs, induced neural stem cells; VPA, Valproic acid.

## Data Availability

No new data were created or analyzed in this study. Data sharing is not applicable to this article.

## Abbreviations

AD, Alzheimer’s disease; aOECs, Activated olfactory ensheathing cells; ASD, Autism spectrum disorder; Aβ, β-amyloid; BBB, Blood–brain barrier; BDNF, Brain-derived neurotrophic factor; BMSCs, bone marrow mesenchymal stem cells; ceRNAs, Competing endogenous RNAs; CHI, Closed head injury; CNS, Central nervous system; Crry, Complement receptor 1-related protein y; CS-A, Chondroitin sulfate; CS-A + NPCs, CS-A-encapsulated NPCs; DLL1, Delta-like ligand 1; EAE, Experimental autoimmune encephalomyelitis; GDNF, Glial cell-derived neurotrophic factor; GM-CSF, Granulocyte-macrophage colony-stimulating factor; ICH, Intracerebral hemorrhage; IGF-1, Insulin-like growth factor-1; IL, Interleukin; IM1-MPs, Inflammatory type 1 mononuclear phagocytes; iPSCs, induced pluripotent stem cells; IVH, Intraventricular hemorrhage; lncRNA, Long non-coding RNA; MCAO, Middle cerebral artery occlusion; MMP, Matrix metalloproteinase; MS, Multiple sclerosis; NGF, Nerve growth factor; NPCs, Neural progenitor cells; NSC, Neural stem cell; NSCs-Sox2, Sox2-overexpressing NSCs; NSPC, Neural stem/progenitor cell; Nurr1, Nuclear receptor-related factor 1; PD, Parkinson’s disease; PHH, Post-hemorrhagic hydrocephalus; SCI, Spinal cord injury; Shh, Sonic hedgehog; TBI, Traumatic brain injury; TGF-β, Transforming growth factor-β; TH, Tyrosine hydroxylase; TLR4, Toll-like receptor 4; TNF-α, Tumor necrosis factor-α; Tregs, Regulatory T cells.
